# Botulism and cavernous sinus thrombosis induced by acute rhinosinusitis: A case report

**DOI:** 10.22088/cjim.9.2.194

**Published:** 2018

**Authors:** Ali Tavassoli, Mahmood Sadeghi, Parviz Amri

**Affiliations:** 1 Clinical Research Development Unit of Babol University of Ayatollah Rouhani Hospital, Babol University of Medical Sciences, Babol, Iran.; 2 Infectious Diseases and Tropical Medicine Research Center, Health Research Institute, Babol University of Medical Sciences, Babol, Iran.; 3 Cancer Research Center, Health Research Institute, Babol University of Medical Sciences, Babol, Iran.

**Keywords:** Acute sinusitis, Botulism, Paralysis

## Abstract

**Background:**

Botulism is an acute and rapidly progressive descending paralytic disease caused by a neurotoxin of clostridium botulinum.

**Case presentation:**

A 28-year-old woman presented with severe generalized ascending symmetrical muscle paralysis. The patient was intubated and transferred to the medical intensive care unit with several symptoms including: severe headache, dysphagia, dyspnea, ptosis, diplopia, and dry mouth. Despite being alert, pupils were bilaterally midriatic and had absent corneal reflux. Pansinusitis was seen in the paranasal sinus scan. At first, the movement of eyelids, head and neck were restored. The movement of the upper limbs (15^th^ day) and chest wall (20^th^ day), abdomen (25^th^ day) and the lower extremities (32nd day) were then gradually restored. On 41^st^ day, the patient was completely disconnected from the ventilator.

**Conclusions:**

Botulism should be a diagnosis in any patient with an acute progressive symmetrical descending paralysis. Sinus mucosal injury (acute sinusitis) can be inoculated with spores of *botulinum.*

Botulism is a rare, serious and progressive descending neuroparalytic disease that is caused by a toxin produced by the *bacterium* *Clostridium botulinum* (1, 2). The toxin blocks the acetylcholine transmission in all ganglionic synapses and neuromuscular junctions may lead to respiratory failure and death (3, 4). There are three major types of botulism; 1) food-borne botulism is caused by eating food that contains toxins; 2) wound botulism is caused by infected wound with C*lostridium* *botulinum*; 3) infant botulism is caused by consuming bacteria-containing particles (4). Inhalation botulism has been identified only in a single outbreak in human but has recently received more attention due to its potential for aerosolized toxin used as a biological weapon (5). Initial diagnosis is based on clinical presentation. A definitive diagnosis is based on signs and symptoms in mice after administration of patient’s serum and demonstration of toxin in the serum (4). We report a case of botulism in a 28-year-old female patient whom we did not initially find any evidence of botulism etiology. However, after thorough investigation, we found that the patient is suffering from acute pansinusitis. 

## Case Presentation

A 28-year-old woman was transported to the emergency unit of Ayatollah Rouhani Hospital in Babol in January 2013 due to generalized muscle paralysis and respiratory failure. The patient underwent intubation and was transferred to the medical intensive care unit.

The patient had a history of severe headache and fever was reported a week ago. She was reported to have had dysphagia, dyspnea, ptosis, diplopia, dry mouth and weakness of extremities 12 hours before respiratory failure. Weakness of muscle initially occurred in the upper extremities and then in the lower extremities. Vital signs include: blood pressure: 130/80 mmHg, heart rate: 120/min, respiratory rate: 12 (on ventilator without any trigger and assist), temperature: 38.2^ oC^ and saturation of peripheral oxygen: 100%.

On examination, the patient was alert; pupils were bilaterally midriatic but with absent corneal reflux absent. There was no papilledema seen using an ophthalmoscope. Swallowing reflex was absent. 

**Figure 1:**
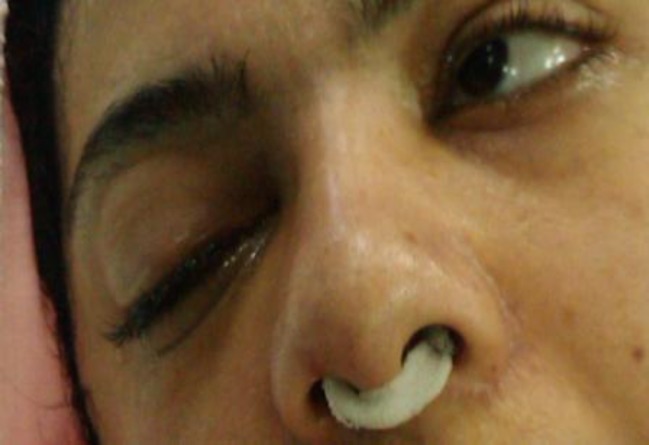
The patient face pre FESS

The patient’s extremities were quadriplegic and are areflexic in all four limbs. There was not any movement on the eyelids, cheeks, chin, head and neck. Facial and frontal folds were completely omitted (figure 1). She had mild abdominal distension.

On respiratory system, the patient had apnea and on ventilator with ACMV, TV=550 ml, RR: 12 / min, PEEP = 3 Cm / H2O, Fio_2_= 50% did not need any assistance (6, 7). Initial tests: WBC: 13500 μL, PLT: 23600 μL , Hb: 11.7 gr/dl, HCT: 34.8%, Bun: 13 mg/dl, Cr: 0.6 mg/dl, BS: 127 mg/dl, Ferritin: 196 ng/mL, Na: 131 mEq/L, K: 3.7 mEq/L, CL: 97 mEq/L, Mg: 1.8 mEq/L, SGOT: 17 U/L, SGPT: 10 U/L, ALP: 176 U/L, PT: 13.2, PTT: 35, INR: 1.3, urine analysis and urine culture were normal.

**Figure 2:**
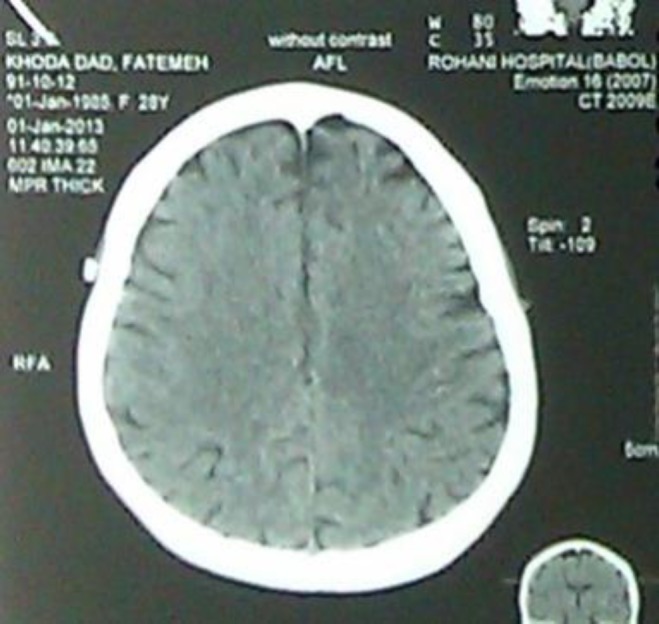
Normal brain CT scan

Biochemical analysis of CSF was normal. In addition, the culture was negative; PCR was negative for mycobacterium tuberculosis. Qualitive test for HSV-I and HSV -2 are negative. Chest x-ray and brain CT scan was normal (figure 2).

**Figure 3:**
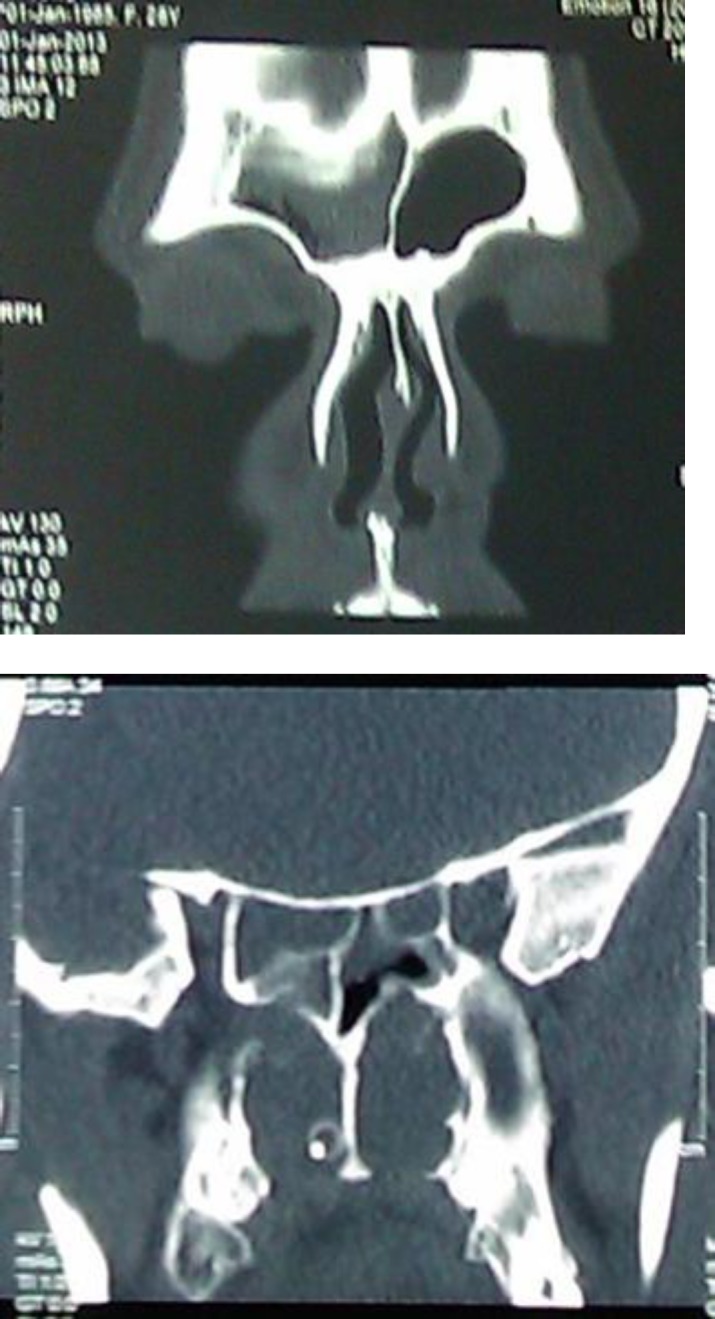
a-Sinusitis in paranasal scan b- Frontal sinusitis

There were pansinusitis in the paranasal sinus scan (Figures 3a and 3b).

Parameters of arterial blood gases in the first hour after the start of mechanical ventilation were, PH: 7.33, PCo2: 47 mmHg, PO2: 119 mmHg, HCO3: 23.7 mmol/L. Arterial blood gas was checked at least once daily during hospitalization and if necessary, was corrected.  Due to the complete relaxation, Botulinum antitoxin was administered. Other treatments include antibiotics for acute sinusitis (amikacin 1 g IV daily, vancomycin 1 g intravenously twice daily, meropenem (IV) 1 g every 8 hours) pantoprazole 40 mg IV twice daily, subcutaneous enoxaparin 40 mg daily. Neurology consultation revealed bilateral exophthalmos, chemosis, proptosis, and lack of vertical and horizontal movement of both eyes. The neurologist diagnosed venous thrombosis based on clinical examination and then started subcutaneous enoxaparin 40 mg twice per day, dexamethasone 8 mg IV every 8 hours and antibiotics. Na, K, Mg, Ca, P were checked in the the ICU, and if necessary, were corrected.

**Figure 4:**
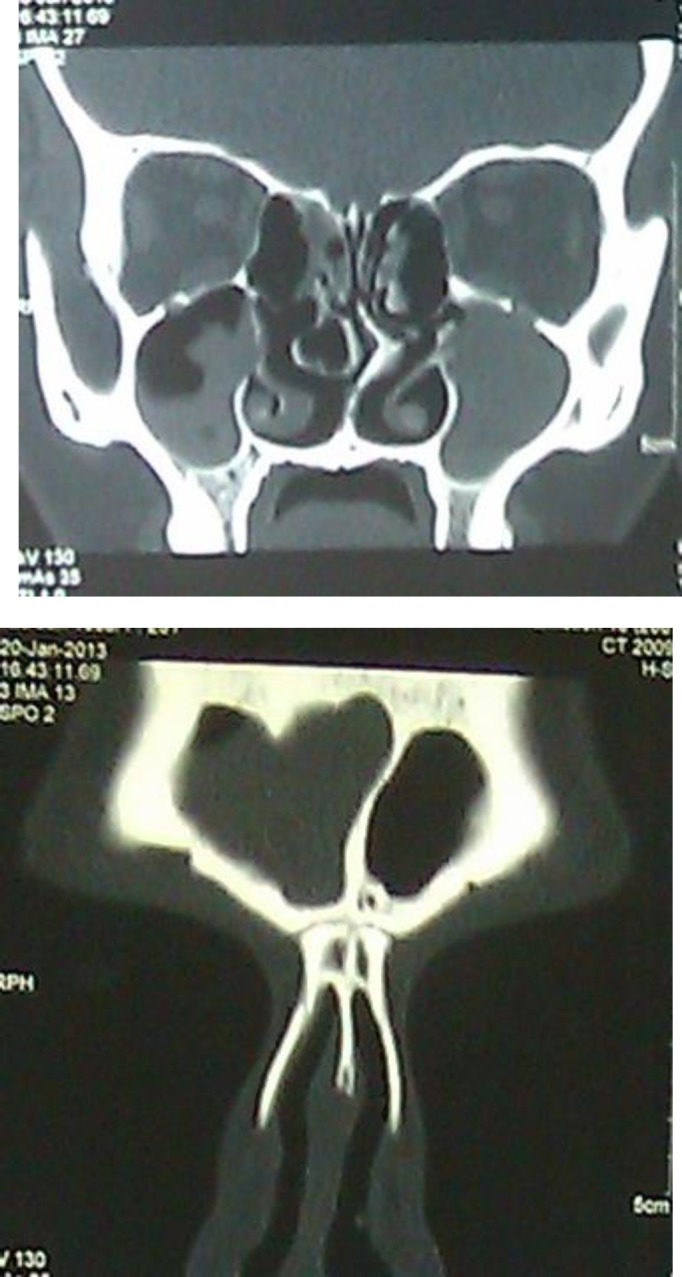
A-Sinuses after FESS, B- Frontal sinus after FESS

On the seventh day of hospitalization, functional endoscopic *sinus* surgery (FESS) was carried out and purulent sinus drainage was evacuated (figures 4a and 4b).

On the ninth day, the first signs of spontaneous respiration of the brain stem (MIP=-2 cm / H20) was observed. Then, the patient's spontaneous breathing was assisted by ventilator. A tracheostomy was performed on the thirteenth day. Then, gradually the movement of the upper limbs (15^th^ day), chest wall (20^th^ day), abdomen (25^th^ day) and the lower extremities (32^nd^ day) were restored.

On the 37^th^ day of admission, the patient tolerated the mode of pressure support ventilation (PSV=14 cm/ H2o). The pressure level gradually decreased based on the patient’s respiratory rate and tidal volume (6, 7). On the 41^st^ day, the patient was completely disconnected from the ventilator and better tolerated T-tube with 5 liters oxygen per minute. Then after 72 hours (44^th^ day), tracheostomy tube was removed. Arterial blood gases were normal during mechanical ventilation. Joint movement and muscular strength gradually were restored after two months of therapy and became relatively normal. After 6 months, muscle strength was completely normal. For now, the patient’s activity and function of other organs are normal.

## Discussion

In this report, we introduced a patient with rapid progressive descending symmetrical paralysis. Botulism is usually considered a foodborne infection. In our patient, there was no evidence of wound and intestinal botulism. However, after evaluating the patient, we found acute sinusitis (based on CT scan) and cavernous sinus thrombosis (according to the neurologist’s consultation). Our observation suggested that botulism was caused by entrance of bacteria in inflamed sinuses and sinus thrombosis cavernous is one of the complications of acute sinusitis.

Ghasemy.et al presented a 33-year-old woman with a diagnosis of botulism associated with venous thrombosis (4). The patient recovered (without intubation) after receiving antitoxin. In contrast to the previous study, our patient did not completely respond to antitoxin. Some differential diagnoses of botulism are Guillain-Barre syndrome, Miller-Fisher syndrome, myasthenia gravis, Eaton- Lambert syndrome, poliomyelitis, and drug intoxications like Mg and atropine (8). Guillain-Barre´ syndrome follows an acute infection, presents in 95% of cases as ascending paralysis. Myasthenia gravis should be considered and the Tensilon test should be administered in case of doubt, even if borderline tensilon test results have been reported for patients with botulism (5). Definitive diagnosis can be possible by demonstration of toxin in the serum but it may be negative despite infection and cannot be conducted in all laboratories (4). Roblot et al. described two cases of mild botulism in patients who inhaled cocaine. Those two patients presented with sinusitis, and, in one case, a sinus aspirate sample grew *Clostridium botulinum* (5). The presentation of bilateral proptosis of the eyes is pathognomic for cavernous sinus thrombosis (9). The cranial nerve palsies are explained by the anatomical course of these nerves as they pass through the vicinity of the cavernous sinus, and this can result in compression of the third, the fourth, and the sixth cranial nerves (9). Despite the absence of magnetic resonance imaging (MRI) which is necessary for the confirmation of diagnosis (because of lack of compatible MRI ventilator in our hospital), cavernous sinus thrombosis was the most likely the diagnosis with this clinical presentation.

In conclusion, despite being a rare disease, botulism should be a diagnosis in any patient with an acute symmetrical descending progressive paralysis. It is important to note that fever and leukocytosis do not rule out the diagnosis of botulism, since it may be accompanied with other diseases such as acute sinusitis and venous sinus thrombosis. Finally, sinus mucosal injury due to acute sinusitis can be inoculated with spores of *botulinum.*
